# Evaluating headache referral trends and practices across different settings in neurology clinics: insights from an international cross-sectional multicenter study

**DOI:** 10.22514/jofph.2025.019

**Published:** 2025-03-12

**Authors:** Hamit Genc, Derya Uluduz, Hayrunnisa Bolay, Betul Baykan, Isin Unal-Cevik, Najib Kissani, Otgonbayar Luvsannorov, Mansoureh Togha, Asena Ayca Ozdemir, Aynur Ozge, on behalf of the Head-MENAA Study Group

**Affiliations:** ^1^Neurology Clinic, Health Application and Research Center, Gaziantep City Hospital, 27470 Gaziantep, Türkiye; ^2^Department of Neurology, Medical Faculty, Istanbul University-Cerrahpaşa, 34098 Istanbul, Türkiye; ^3^Department of Neurology and Algology, Faculty of Medicine, NOROM, Gazi University, 06560 Ankara, Türkiye; ^4^Neurology Clinic, EMAR Medical Center, 34367 Istanbul, Türkiye; ^5^Department of Neurology, Headache and Pain Unit, Faculty of Medicine, Hacettepe University, 06100 Ankara, Türkiye; ^6^Neuroscience Research Laboratory in Marrakesh Medical School, Department of Neurology, Cadi Ayyad University, 40000 Marrakech, Morocco; ^7^Department of Neurology, Mongolian National University of Medical Sciences, 14210 Ulaanbaatar, Mongolia; ^8^Department of Neurology, Sina Hospital, School of Medicine, Tehran University of Medical Sciences, 1417613151 Tehran, Iran; ^9^Department of Headache, Iranian Center of Neurological Research, Neuroscience Institute, 1983969367 Tehran, Iran; ^10^Department of Medical Education, Mersin University, 33343 Mersin, Türkiye; ^11^Department of Neurology, Faculty of Medicine, Mersin University, 33343, Mersin, Türkiye

**Keywords:** Referred patients, Headache severity, Headache frequency, Lifetime pain duration, Primary headaches, Secondary headaches, Neurology clinic, Emergency service, Specialty clinic, Private office

## Abstract

**Background**: Misdiagnoses often lead to suboptimal therapeutic approaches, making early and accurate diagnoses by experts crucial for effective headache management. This study primarily aims to investigate the referred patient profiles with headaches to optimize diagnostic and referral approaches. **Methods**: In this cross-sectional multicenter international study, sixty-nine neurologists from 13 countries evaluated headache patients referred to neurology clinics (NCs). Researchers recruited patients on different weekdays selected by the research randomizer program for five consecutive weeks in April and May 2022. The clinicians collected data on various factors such as age, sex, headache characteristics and accompanying symptoms using the Head-MENAA study questionnaire and the International Classification of Headache Disorders-3 (ICHD-3) criteria. Patients were grouped according to the settings as emergency services (ESs), other specialty clinics (OSCs) and private offices (POs) in which they were evaluated. **Results**: A total of 3722 individuals out of 12043 evaluated in NCs had headache complaints. Among them, 15.07% consisted of patients referred to neurology by these three different settings. 14.8% of them were referred from ESs, 16.58% from OSCs, and 68.64% were applied to POs. While there was not a significant difference between groups regarding the mean age, the proportion of male patients in the ESs (49.4%) was higher than those in OSCs (26.9%) and POs (23.1%) (*p* < 0.001). Headache severity was higher in the ESs and POs than in the OSCs and Neurology Outpatient Clinics (NOCs) (*p* < 0.001). Primary headaches were the reason for consultation in 89.2% of patients in the ESs, 90.3% of patients in OSCs and 93.5% of patients in POs, migraine without aura being the most common headache type in all groups. **Conclusions**: This study suggests that preferences for admission and referral may vary based on demographic characteristics, types and severity of the headache, as well as accessibility and availability of different settings.

## 1. Introduction

Headache is a disorder that is often underdiagnosed and undertreated [1], can cause significant disability [2], and is frequently encountered in neurology practice [3]. While a large proportion of headache patients do not seek medical attention [4, 5], some lack access to healthcare providers who are knowledgeable and trained in headache management [6]. This condition has become a common problem in primary care, leading to referral to neurology clinics (NCs).

The main reasons for referral to NC include diagnostic uncertainty, indecision for treatment, patient anxiety, as well as inadequate sources for tests to identify secondary causes [7, 8, 9]. Referral of selected patients is of clinical importance because the gold standard for diagnosing headaches is a face-to-face interview and examination by a neurologist [10]. Although neurologists are essential in providing appropriate diagnosis and treatment, the main problem is the number of neurologists per capita worldwide. Neurologist/population ratios vary significantly by geographic region. For example, in the United States and Europe, the estimated ratio of neurologists to the population ranges from 0.56 to 12.3 per 100,000 population [11], while in Africa, this value is only 0.043 [12].

In the light of these data, we aimed to investigate the profiles of referred headache patients to understand the demographics, clinical features and reasons for the referral—(including examples like the need for an accurate and definitive diagnosis, scarce response to current treatments, the need for comprehensive evaluation of secondary headache causes, headache severity, and frequency that seriously affect patients’ quality of life, non-compliance with treatment or consideration of alternative treatment methods due to side effects) to determine the unmet needs of patients and to develop more effective referral algorithms, education and strategies tailored for these needs.

## 2. Materials and methods

### 2.1 Study design

This follow-up study of a former one designed as a multinational and multicenter cross-sectional study was conducted five weeks from 01 April to 16 May 2022. During this period, 69 neurologists included patients on a designated day each week. The patient admission days were picked using the “Research Randomizer Program”. Neurologists evaluated all patients in detail, and all investigators received structured training on the International Classification of Headache Disorders-3 (ICHD-3) criteria [13] to increase study reliability.

All volunteers aged 18 years and older with headaches as the primary cause for admission were included in the study. It excluded patients younger than 18, without headaches, and did not agree to participate in the study. Online informed consent forms were obtained from volunteers who participated in the study. A structured questionnaire (Head-MENAA Study Questionnaire—**Supplementary material 1**) was administered to participants, including demographic information and headache characteristics questions [3]. Headache severity was evaluated by a numbered visual scale (NVS). Neurologists coded headache subtypes according to ICHD-3 criteria [13] in the same questionnaire.

Patients referred to NC were evaluated in this substudy of the Head-MENAA study. Patients were grouped into three different settings as referred from: (a) emergency services (ESs), (b) other specialty clinics (OSCs), and (c) those assessed in private offices (POs). The study population consisted of participating regions from the Middle East (Egypt, Iran), North Africa (Ivory Coast, Chad, Senegal, Sudan, Ethiopia, Morocco), and Asia (Türkiye, Turkish Republic of Northern Cyprus, Azerbaijan, Tatarstan, Mongolia) (Fig. 1). Therefore, the name of the study was determined as “Head-MENAA” (Middle East, North Africa, Asia) using the initials of these regions. The study coordinator obtained Ethics Committee approval from the Clinical Research Ethics Committee, University of the Health Sciences, Van Training, and Research Hospital, Van, Türkiye (Decision no: 2022/05–01, Date: 02 March 2022).

**Fig. 1.** The distribution of referred patients according to the three different clinical settings in neurology. (NOCs: Neurology Outpatient Clinics, *Only Tatarstan region joined the study from Russia, **#**The countries are listed in alphabetical order.)

### 2.2 Statistical analysis

Normality control of continuous variables was established with the Shapiro-Wilk test. Parametric tests were used for the variables that fit the normal distribution, and non-parametric tests were used for the ones that did not. One-way Analysis of Variance (ANOVA) and Kruskal Wallis tests were used to carry out the comparative analysis of more than two groups. In addition, the Tukey test was used as the *post-hoc* test. The chi-square test was applied to analyze categorical data. The statistical significance level was taken as *p* < 0.05. The analysis of the data was carried out through the TIBCO Statistica 13.5.0.17 program (TIBCO Software Inc., Palo Alto, CA, USA).

## 3. Results

A total of 3722 out of the 12,043 patients evaluated in NCs on selected days had reported headache complaints. 15.07% of the patients presenting with headache complaints were referred to NCs. 14.8% (83/561) of these referred patients were consulted from ESs, 16.58% (93/561) from OSCs and 68.64% (385/561) were applied directly to Pos (Fig. 1).

The proportion of male patients in the ESs (49.4%) was higher than the proportion of male patients in OSCs (26.9%), POs (23.1%) and in all NOCs (25.2%) (*p* < 0.001). There was no significant difference in mean age between these groups (*p* = 0.266). Headache severity was higher in ESs and POs than in OSCs and NOCs (*p* < 0.001). Lifetime pain duration was shorter in patients consulted by ESs, and headache frequency was lower than in patients consulted by OSCs. In POs, lifetime pain duration was more prolonged, and headache frequency was higher than in patients consulted from ESs and OSCs (*p* < 0.001). When patients were evaluated in the NOCs compared with those consulted from ESs and OSCs, there was prolonged lifetime pain duration and increased headache frequency (*p* < 0.001). Headache severity was higher in patients in NOCs than in patients consulted from OSCs (*p* < 0.001) (Table 1).

**Table 1.** Demographic and headache characteristics according to the examination and referral settings.

	Emergency services (^a^)	Other specialty clinics (^b^)	Private offices (^c^)	Neurology outpatient clinics (^d^)	*p* value
Gender (F/M)	1.02^a^	2.72^b^	3.33^b^	2.97^b^	<0.001*
Age (Mean ± SD)	44.09 ± 14.00	42.47 ± 16.98	42.72 ± 12.43	42.89 ± 15.00	0.266^†^
Lifetime pain duration (yr) (Mean ± SD)	0.64 ± 2.52	2.18 ± 4.07^a^	11.84 ± 11.65^abd^	5.15 ± 7.68^ab^	<0.001^†^
Severity of headache (NVS) (Median ± SD)	8 [6–9]^bd^	6 [5–8]	8 [7–8]^bd^	7 [6–8]^b^	<0.001ꭞ
Frequency of headache (Median ± SD)	2 [1–5]	4 [2–13.5]	12 [6–25]^abd^	8 [3–20]^ab^	<0.001ꭞ

SD: Standard deviation; F: Female; M: Male; yr: years; NVS: Numbered visual scale; *Chi-Squared test, ^†^Independent Sample t test, ꭞMann Whitney U test (Each superscript letter denotes a subset of categories whose column proportions/means differs significantly from each other at the significant <0.05 level). a: Emergency services; b: Other specialty clinics; c: Private offices; d: Neurology outpatient clinics.

The reason for consultation was primary headache in 89.2% of patients in the ESs, 90.3% of patients in OSCs, and 93.5% of patients in POs. The most common headache diagnosis in ESs, OSCs, and POs was migraine without aura, 56.6%, 59.1% and 55.8%, respectively. The tension-type headache (TTH) rate in patients consulted from OSCs was higher than in POs (*p* = 0.047). There was no difference in secondary headaches in patients referred from the ESs (36.1%), OSCs (33.3%) and POs (30.6%). Headache attributed to psychiatric disorders was observed at a higher rate in the ESs than in POs (*p* = 0.028) (Table 2).

**Table 2.** Distribution of headache types based on the clinic of referral.

International Classification of Headache Disorders-3 (ICHD-3) Criteria	Emergency services (^a^) n (%)	Other specialty clinics (^b^) n (%)	Private offices (^c^) n (%)	Total n (%)	*p* value
Primary Headaches					
1. Migraine	58 (69.9)	58 (62.4)	279 (72.5)	395 (70.4)	0.159
2. Tension-type headache (TTH)	20 (24.1)	33 (35.5)^c^	89 (23.1)	142 (25.3)	0.047
3. Trigeminal autonomic cephalalgias (TACs)	6 (7.2)	3 (3.2)	19 (4.9)	28 (5.0)	0.475
4. Other primary headache disorders	1 (1.2)	3 (3.2)	10 (2.6)	14 (2.5)	0.674
Secondary Headaches					
5. Headache attributed to trauma or injury to the head and/or neck	0 (0.0)	1 (1.1)	3 (0.8)	4 (0.7)	0.673
6. Headache attributed to cranial or cervical vascular disorder	4 (4.8)	3 (3.2)	15 (3.9)	22 (3.9)	0.862
7. Headache attributed to non-vascular intracranial disorder	5 (6.0)	4 (4.3)	16 (4.2)	25 (4.5)	0.754
8. Headache attributed to a substance or its withdrawal	8 (9.6)	8 (8.6)	49 (12.7)	65 (11.6)	0.448
9. Headache attributed to infection	2 (2.4)	0 (0.0)	6 (1.6)	8 (1.4)	0.374
10. Headache attributed to disorder of homeostasis	3 (3.6)	5 (5.4)	12 (3.1)	20 (3.6)	0.573
11. Headache or facial pain attributed to disorder of the cranium, neck, eyes, ears, nose, sinuses, teeth, mouth or other facial or cervical structure	6 (7.2)	8 (8.6)	19 (4.9)	33 (5.9)	0.343
12. Headache attributed to psychiatric disorder	5 (6.0)^c^	3 (3.2)	5 (1.3)	13 (2.3)	0.028
Neuropathies & Facial Pains and Other Headaches					
13. Painful lesions of the cranial nerves and other facial pain	1 (1.2)	4 (4.3)	11 (2.9)	16 (2.9)	0.468

*p: Chi-Squared test (Each superscript letter indicates a subset of categories with column proportions that differ significantly from each other at the p > 0.05 level). a: Emergency services; b: Other specialty clinics; c: Private offices.*

When headache subtypes were examined separately, frequent episodic TTH was more frequently referred from OSCs than in POs (*p* = 0.016). Probable TTH was more frequently referred from ESs and OSCs than in POs (*p* = 0.008). Short-lasting unilateral neuralgiform headache attacks (*p* = 0.038) and probable trigeminal autonomic cephalalgias (TACs) (*p* = 0.038) were observed more frequently in patients referred from ESs, whereas hemicrania continua (*p* = 0.026) was observed more frequently in patients referred from OSCs than in POs. Headaches attributed to cerebral venous thrombosis (CVT) were more frequently referred from ESs than POs (*p* = 0.003). Headaches attributed to somatization disorder were observed more frequently in patients referred from ESs than in POs (*p* = 0.050). Medication-overuse headache (MOH) was the most common subtype of secondary headaches. It was detected in 9.6% of referred patients from ESs, 8.6% from OSCs and 12.2% from POs. There was no disparity in the frequency of other headache subtypes regarding the regions to which the patients were referred.

## 4. Discussion

Our study is the first to examine the differences in admission and referral patterns across various NC settings concerning age, gender, headache frequency and severity, lifetime pain duration, and headache diagnoses based on ICHD-3 criteria [13]. Although no significant differences were found in the mean age between groups, the proportion of male patients was notably higher in the ESs. The findings also reveal higher headache severity in the ESs and POs,

along with longer pain duration and more frequent headaches in the POs. Furthermore, the high prevalence of primary headaches, expressly migraine without aura, highlights the importance of addressing these conditions. The prevalence of MOH as the most common secondary headache subtype further accentuates the need for effective prevention strategies. Data were analyzed from 13 distinct geographic regions, reflecting notable variations in health systems. According to the World Bank Group country classifications by income level, Cyprus is placed in the high-income group; Azerbaijan, Türkiye and Russia are in the upper-middle income group; Egypt, Iran, Ivory Coast, Mongolia, Morocco and Senegal are in the lower-middle income group; and Chad, Ethiopia and Sudan are in the low-income group [14]. When we look closely at some different countries’ health systems, in Türkiye, there are centers for headache treatment, such as free public hospitals, university hospitals and private hospitals. Unfortunately, patients can challenge long waiting times in the public sector [15]. In Russia, there are improving efforts to increase migraine awareness, ease the use of ICHD criteria, ensure the dissemination of territorial headache centers, and provide communication between public centers and the Ministry of Health [16]. Despite growing efforts to facilitate health care in low- and middle-income countries, it is essential to remember that there are significant challenges, such as awareness for headache disorders, undersupply of education, access to care, and economic barriers in the diagnosis-treatment process, and insufficient health policies [17].

### 4.1 Consultation patterns of neurologic care for headaches

Many headache patients do not seek medical attention for their symptoms [5]. For example, headaches in Africa are primarily self-treated, mainly due to limited primary care and neurological consultation opportunities [6]. In addition to patients being reluctant to seek medical attention for their headaches, some patients lack access to healthcare providers due to socioeconomic, time, and transportation reasons [6, 18]. On the other hand, in many countries, healthcare services cannot meet the needs of many patients with headaches, even when they seek medical attention, due to the lack of neurologists and diagnostic support [11, 12, 19, 20, 21, 22]. Therefore, improving knowledge and developing effective referral systems may be essential in headache management.

In many regions, headaches are managed in primary care [7, 19]. In some regions, direct referrals can be made to neurology care or private headache clinics [23, 24]; in countries such as Spain and Norway, headache clinics are designed on a mixed referral model based on collaboration with primary care [25, 26].

Headaches are among the most frequent problems in primary care and the foremost reason for neurological consultations [25]. A comprehensive interview and physical examination of the patient can furnish the information needed to establish a correct headache diagnosis. It can also prevent unnecessary neuroimaging tests by allowing the exclusion of structural secondary causes [26]. However, these patients with primary headaches are often triaged at low priority and must be adequately treated in primary care [7]. Another area for improvement is patients’ difficulties receiving correct diagnosis and adequate treatment in primary care settings such as the ESs. In one study, only 18% of patients received analgesic treatment upon discharge from the ESs, leading to repeated emergency visits [27]. The unmet need for satisfactory acute headache treatment is the leading reason for ESs visits for primary headache patients [28]. The applicability of the ICHD criteria [13] in an ES is hampered by crowded ESs, nonlinear acceptance of severe cases, time constraints for obtaining a detailed history, and difficulty obtaining a history from the patient during the painful period, besides the lack of knowledge and training on the headache diagnosis [27, 29]. In a way that supports this, a study conducted in 2007 demonstrated that the application of ICHD criteria [13] in the ESs allowed correct diagnosis in only two-thirds of patients [30]. Another major problem is the excessive and inappropriate use of neuro-imaging methods in primary headaches due to several reasons, such as malpractice risk and patient anxiety [24]. Adopting suitable approaches in primary care and establishing a structured referral chain can support the efficient use of resources, the provision of correct diagnosis and treatment, and the determination of appropriate priorities among patients to be referred.

The referral decision is influenced by many factors, including diagnostic uncertainty, treatment decisions, patient anxiety and the referrer’s suspicion of underlying secondary causes needing neurologic care [8, 9]. Sources of referral to neurology include general practitioners, other specialists (especially neurosurgeons), other healthcare personnel, hospital staff and family members, in addition to their own applications [25, 31, 32, 33, 34]. The rates of referred patients may vary according to healthcare systems. For example, as mentioned above, in regions where the referral system is mandatory, the rate of patients referred to neurology by primary care is 55.3–71.7% [25, 33], while in areas where the referral system is not mandatory, this rate drops to 23–30% [31, 34]. In our study, patients from the ESs and OSCs were referred to neurology by general practitioners (GPs) and other specialists, reflecting their real-life experiences.

In a study conducted in the USA, the main complaint of 2.2–3.2 out of every 100 people who applied to ESs was headache [35, 36]. In a study conducted in Zagreb, one in five patients who applied to the ESs had a headache, three-quarters of which were primary headaches [37]. The rate of neurology consultations for headaches in patients who applied to the ESs differs between centers. In a study conducted in Türkiye, 0.83% of patients who applied to the ESs consulted neurology, but the headache was not among the reasons [38]. In contrast, in another study conducted in Türkiye, headache was the second most common reason for consultation (13.5% of consulted patients) [39]. These different patterns reflected that the referral system is not structured and its reasoning is not well-established.

In a study conducted in the UK, which uses the GP system more effectively, 2.1 out of 100 patients diagnosed with headaches in general practice were referred to neurology [21]. The rate of headache among patients referred to NOCs was 30.04% in our study, which was similar to studies in Cameroon (31.9%) [40] and Ecuador (33.2%) [34]. In contrast, in a study conducted in New Zealand, 69% of patients referred to neurology were headache patients [7]. Among the patients evaluated for headaches in our study, those referred from the ESs constituted 0.02% of all patients admitted for any reason, those referred from OSCs constituted 0.03%, and those evaluated in POs constituted 0.1%.

### 4.2 Demographic characteristics of the patients admitted to different settings

A study in the United Kingdom reported that young women had the highest headache consultation rates [21]. Indeed, in our research, there was a similar female predominance in areas other than the ESs. Our study also found that the proportion of male patients admitted to the ESs was significantly higher than in other clinical settings (Table 1). While 49.4% of patients referred from the ESs were male, this rate was 35.5% in a study conducted in Zagreb [37]. This may be due to men being less likely to seek routine medical care, longer waiting times to access a neurologist, socioeconomic reasons, and the fact that ESs are more accessible for pain management. According to one study, men are 60% less likely to seek medical attention than women [41]. Male patients are thought to delay seeking advice for headaches and use more over-the-counter analgesics to manage this condition. Indeed, a higher incidence of MOH was observed in men in one series [42]. In addition, a study conducted in Ireland showed that longer waiting times for appointments at the NCs and economic reasons were tribulations in headache management [43]. The literature still needs to learn the effects of many factors that may affect differences in gender preferences, such as sociocultural, economic, psychological and race.

There was no difference in mean ages between headache patients consulted from ESs and OSCs and those evaluated in POs, as seen in Table 1. Similar rates were also obtained in other studies. For example, the mean age of patients consulted from a private headache clinic was 42 years [25]. In another study evaluating patients referred from primary care to neurology, it was 48.5 [29]. Headache severity was higher in patients referred from ESs (7.48 ± 1.86), as expected, and also for those evaluated in POs (7.69 ± 1.3) compared to those admitting to NOCs (6.98 ± 1.74) and consulted from OSCs (6.32 ± 1.75). The headache frequency and lifetime pain duration of the patients evaluated in our study can be listed from the lowest to highest as ESs < OSCs < POs (Table 1). Similar to our study, the pain intensity of patients assessed in the ESs in Italy was also high (8.8 ± 1.6) [26]. It seems that individuals whose headaches are not severe enough and can tolerate headache symptoms generally do not admit to the ESs [5]. Since the neurologists working in POs in our study typically consist of physicians specializing in headaches, this group’s pain intensity, frequency, and lifetime pain duration were higher, probably because resistant headache cases are referred to these specialists.

### 4.3 Headache types and subtypes of the patients admitted to different settings

In our study, the primary headache rates in patients evaluated in the ESs, OSCs and POs were 89.2%, 90.3%, and 93.5%, respectively. In studies, the rate of headache among patients presenting to the ESs is around 2.2–3.2% [22, 35, 37]. In another study evaluating ESs patients in Ireland, the rate of primary headaches was 31% [22]. In Ecuador, 72% of headache patients consulting neurology were diagnosed with primary headache [34]. In our study, the rate of migraine headaches among primary headaches in patients assessed in the ESs, OSCs, and POs was 69.9%, 62.4%, and 72.5%, respectively, while the rate of TTH was 24.1%, 35.5% and 23.1% (Table 2). Approximately 50% of migraine patients and 16% of TTH patients consult a general practitioner for headache complaints [44]. In a study conducted in the USA, 63.5% of headaches in ESs were caused by migraine and vascular headaches, while 3.4% were caused by TTH [35]. Although TTH constitutes the most significant percentage of total headaches in population-based studies, migraine is observed at a higher rate in primary care [45]. The reason for the higher rate of migraine may be due to increased pain intensity, longer attack duration, and increased aura frequency [27]. In our study, patients with frequent episodic TTH were referred to NOCs more frequently from OSCs than POs.

In contrast, patients referred with probable TTH to NOCs were more often from ESs and OSCs than POs. When all these findings are evaluated together, it can be concluded that primary headaches (especially TTH) are not sufficiently recognized and treated in the settings participating in the study (especially by general practitioners and other specialists). At the same time, this situation may increase the burden on the health system and neurologists. A study conducted in low and middle-income countries showed that TTH needs to be better recognized, supporting this conclusion [46]. Another review suggested that there is an unmet need for effective diagnosis, treatment and management of migraine in East Asia [4]. Short-lasting unilateral neuralgiform headache attacks and probable TACs were more common in patients referred from ESs. Hemicrania continua was more common in patients referred from OSCs than in POs. The reason for this difference, which may prompt emergency visits, is that TACs are characterized by severe pain that awakens from sleep and has pulsatile/sharp quality.

Our study demonstrated that the secondary headache rates of patients evaluated in the ESs, OSCs, and POs were very similar, 36.1%, 33.3% and 30.6%, respectively. Although secondary headaches were more common in the ESs than in OSCs, this was not statistically significant. On the contrary, in a study evaluating patients in the ESs, 15% of patients with headaches had serious secondary causes [22]. Secondary headaches were observed in 21% of patients consulted with headaches to neurology in Ecuador [34]. Remarkably, we found that two diverse causes, “Headache attributed to CVT” and “Headache attributed to somatization disorder,” were observed more frequently in patients referred from the ESs than those referred from POs (Table 2). The most common neurological causes of referral within somatic symptoms are conversion disorders, non-epileptic attacks, and chronic benign headaches [47]. Somatic symptoms are widespread in patients with chronic migraine and chronic daily headaches, especially if these patients have severe headaches, anxiety or depression [48]. The perception of pain during a headache attack and the presence of stressful events and/or psychiatric comorbidities such as anxiety or depression may explain the decision to seek medical help in ESs [49, 50]. A study of adult patients in ESs in the UK found a surprisingly low rate of somatization and somatoform disorders of 3.8% [51]. Although it is unknown how high this rate is, the true ESs prevalence lies somewhere between this figure and the rates seen in primary care and outpatient clinics. Another exciting study result is the higher prevalence of CVT in patients referred from the ESs, possibly because headaches in CVT are acute and more severe [52]. No significant difference was found between the groups for other headache subtypes in which acute and severe headache was observed. This may be because they were mortal, clinical urgency caused neglect to question the headache symptom, and these patients are consulted OSCs, such as neurosurgery. For instance, as seen in Table 2, any patients with headaches attributed to trauma or injury to the head and/or neck were not consulted by neurologists by the ESs. Another striking factor among the secondary headache causes in the study was the intensity of MOH (Table 2). The risk of MOH increases as the number of unsuccessful treatments and doctors consulted increases. Another issue is the recent adoption of the approach of taking medications as early as possible during a migraine attack. This approach increases the likelihood that the patient will frankly take more medicines than necessary and may pave the way for the development of MOH [53]. Chagas *et al.* [42] reported in their study that 55% of headache patients referred by primary and secondary health care used medications prescribed by a doctor, while 34% self-medicated. Another study found that 13.3% of patients with TTH had MOH and that combined analgesics were preferred due to their popularity, availability without a prescription, and being cheap and readily available [54]. Therefore, in regions where the demand for neurology consultation is high, overuse of simple analgesics in patients who cannot access appropriate diagnosis and prophylactic treatment may increase the risk of MOH [7]. The most effective way to prevent MOH is to specify patients at risk and educate them about acute medication use [55].

## 5. Conclusions

In conclusion, our study examined headache patients referred from ESs and OSCs and evaluated in POs. The proportion of male patients was higher in patients referred from ESs. Pain severity was higher in patients referred from ESs and evaluated in POs. Headache frequency was low to high in the ESs, OSCs and POs. The same order was valid for lifetime pain duration from shorter to longer. Primary headache was detected in 92.3% of referred patients, and secondary headache was detected in 31.9%. The most common headache in all areas examined was migraine without aura. While TTH headache was observed more in patients referred from OSCs than in POs, headache due to psychiatric disorders was observed more in patients referred from ESs than in POs. While the frequency of MOH did not differ between the groups, it is noteworthy because it is the most common secondary headache. This study has shown that preferences for headache treatment can change according to personal characteristics, headache type/severity and accessibility and availability of treatment options. In addition, our study may contribute to more effective disease control strategies by identifying critical vital points and potential difficulties.

## 6. Highlights

• Since headache is one of the most common complaints encountered in primary care, providing regular and up-to-date medical education to healthcare professionals (physicians, trainees and other healthcare professionals) in this field can significantly improve the quality of neurological care.

• Informing patients and healthcare professionals about headaches, care areas of neurology and the proper use of analgesics can diminish redundant healthcare visits and provide a more effective treatment process.

• A structured referral system is crucial for timely referral of picked patients and shortening referral times.

## 7. Limitations

• More countries and centers in relevant regions are required to expand the study’s representative power.

• The validity and reliability of the Head-MENAA questionnaire used in the study had not been tested before.

• Although it is disfavorable that factors such as race, sociocultural level and education that may influence the analysis were not questioned in the study, the individuals included in the survey are thought to represent ethnicity in the relevant regions generally.

• Given that our research is hospital-based, it is crucial to recognize the inappropriateness of generalizing its results to the broader population.

## Supplementary Material

Supplementary material associated with this article can be found, in the online version, at https://files.jofph.com/ files/article/1899719818549837824/attachment/ Supplementary%20material.zip.




