# Supplementary material for: Evaluating headache referral trends and practices across different settings in neurology clinics: insights from an international cross-sectional multicenter study
Source: J Oral Facial Pain Headache. 2025 Mar 12;39(1):187–95. doi: 10.22514/jofph.2025.019 (PMC11934733; doi:10.22514/jofph.2025.019)
Supplement: Supplementary file 2 [file Supplementary-material-2.docx]

Supplementary material 2

| **Name** | **Email** | **Affiliations** | **Zip code** | **City** | **Country** |
| --- | --- | --- | --- | --- | --- |
| Melike Cakan | melikecakan@gmail.com | Hacettepe University Faculty of Medicine, Department of Neurology | 06100 | Ankara | Türkiye |
| Yared Mamushet Yifru | yared_mty@yahoo.com | Addis Ababa University, College of Health Sciences, Tikur Anbessa Hospital | 1176 | Addis Ababa | Ethiopia |
| Dereje Melka | m.dereje@yahoo.com | Addis Ababa University, College of Health Sciences, Tikur Anbessa Hospital | 1176 | Addis Ababa | Ethiopia |
| Binyam Alemayehu | binyam.a7@gmail.com | Addis Ababa University, College of Health Sciences, Tikur Anbessa Hospital | 1176 | Addis Ababa | Ethiopia |
| Michael Tesfaye Ketema | michaeltesfaye78@gmail.com | Addis Ababa University, Tikur Anbessa Hospital | 1171 | Addis Ababa | Ethiopia |
| Ramez Reda Moustafa | ramezm@msn.com | Ain Shams University and Neuromed Clinics | 11566 | Cairo | Egypt |
| Ahmed Gomaa Nowar | gomaaa550@gmail.com | Ain Shams University and Neuromed Clinics | 11566 | Cairo | Egypt |
| Etedal Ahmed A. Ibrahim | eetedalibrahim@yahoo.com | Al Neelain University, Faculty of Medicine, The National Centre for Neurological Science | 11111 | Khartoum | Sudan |
| Merve Onerli Yener | onerlimerve@gmail.com | Ankara Bilkent City Hospital, Neurology Clinic | 06800 | Ankara | Türkiye |
|  |  | Migraine Foundation Australia | 3033, | Melbourne | Australia |
| Ruhsen Ocal | ruhsenocal@yahoo.com | Antalya Training and Research Hospital, Neurology Clinic | 07100 | Antalya | Türkiye |
| Nermin Tepe | tepenermin@gmail.com | Balikesir University Faculty of Medicine, Department of Neurology | 10145 | Balikesir | Türkiye |
| Esra Aciman Demirel | esraaciman@yahoo.com | Bulent Ecevit University Faculty of Medicine, Department of Neurology | 67630 | Zonguldak | Türkiye |
| Sibel Cekic | sibelozdemir.2701@gmail.com | Bursa Dr Ayten Bozkaya Spastic Children’s Hospital and Rehabilitation Center | 16250 | Bursa | Türkiye |
| Aysin Kisabay Ak | aysinkisabay@hotmail.com | Celal Bayar University Faculty of Medicine, Department of Neurology | 45030 | Manisa | Türkiye |
| Fatih Celik | fatihcelik9555@gmail.com | Celal Bayar University Faculty of Medicine, Department of Neurology | 45030 | Manisa | Türkiye |
| Agbo Panzo Segla Achi Cedric | cedric_agbo@yahoo.fr | Center Hospital Universal Cocody | BP v 13 | Abidjan | Ivory Coast |
| Valeeva Kadria Gumanovna | valeevakadria@yandex.ru | City Clinical Hospital | 420055 | Kazan | Tatarstan |
| Ahmet Evlice | aevlice@yahoo.com | Cukurova University Faculty of Medicine, Department of Neurology | 01360 | Adana | Türkiye |
| Seck Lala | lalasec@yahoo.fr | Fann hospital of Dakar | 10700 | Dakar | Sénégal |
| Doga Vuralli | dogavuralli@gazi.edu.tr | Gazi University Faculty of Medicine, Department of Neurology | 06500 | Ankara | Türkiye |
| Demet Seker | demet.seker@giresun.edu.tr | Giresun University Faculty of Medicine, Department of Neurology | 28000 | Giresun | Türkiye |
| Husniye Aylin Hakyemez | husniyeaylin@yahoo.com | Giresun University Faculty of Medicine, Department of Neurology | 28000 | Giresun | Türkiye |
| Vedat Ataman Serim | atamanserim@gmail.com | Giresun University Faculty of Medicine, Department of Neurology | 28000 | Giresun | Türkiye |
| Dilek Agircan | d_agircan@hotmail.com | Harran University Faculty of Medicine, Department of Neurology | 63290 | Sanliurfa | Türkiye |
| Tulin Gesoglu Demir | drtulindemir@gmail.com | Harran University Faculty of Medicine, Department of Neurology | 63290 | Sanliurfa | Türkiye |
| Buse Rahime Hasirici Bayir | busehasirci@yahoo.com | Istanbul Haydarpasa Training and Research Hospital, Neurology Clinic | 34674 | Istanbul | Türkiye |
| Burcu Polat | burcupolat@medipol.edu.tr | Istanbul Medipol University, School of Medicine | 34815 | Istanbul | Türkiye |
| Abdulkadir Ermis | kadirermis1@gmail.com | Istanbul Medipol University, School of Medicine | 34815 | Istanbul | Türkiye |
| Elmir Khanmammadov | dr.elmir.khan@gmail.com | Istanbul Medipol University, School of Medicine | 34815 | Istanbul | Türkiye |
| Tugba Okluoglu | okluoglutugba@hotmail.com | Istanbul Training and Research Hospital, Neurology Clinic | 34098 | Istanbul | Türkiye |
| Ozgu Kizek | ozgukizek@gmail.com | Istanbul University, Istanbul Faculty of Medicine, Department of Neurology | 34093 | Istanbul | Türkiye |
| Esme Ekizoglu | esmeekizoglu@yahoo.com | Istanbul University, Istanbul Faculty of Medicine, Department of Neurology | 34093 | Istanbul | Türkiye |
| Elif Kocasoy Orhan | elifkorhan@gmail.com | Istanbul University, Istanbul Faculty of Medicine, Department of Neurology | 34093 | Istanbul | Türkiye |
| Sibel K. Velioglu | sibelkvelioglu@hotmail.com | Karadeniz Technical University (KTU), Faculty of Medicine, Neurology Department Clinical Neurophysiology Unit | 61080 | Trabzon | Türkiye |
| Oznur Kirbasoglu | oznur.55@hotmail.com | Karadeniz Technical University (KTU), Faculty of Medicine, Neurology Department Clinical Neurophysiology Unit | 61080 | Trabzon | Türkiye |
| Samiye Ulutas | uysal.samiye@gmail.com | Kartal Dr. Lutfi Kirdar City Hospital, Neurology Clinic | 34865 | Istanbul | Türkiye |
| Tulin Akturk | tulin_birlik@hotmail.com | Kartal Dr. Lutfi Kirdar City Hospital, Neurology Clinic | 34865 | Istanbul | Türkiye |
| Birsel KUL | drbirselkurt_66@hotmail.com | Kastamonu Training and Research Hospital, Neurology Clinic | 37100 | Karabuk | Türkiye |
| Sibel Canbaz Kabay | scanbazkabay@yahoo.com | Kutahya Health Sciences University, Department of Neurology | 43100 | Kutahya | Türkiye |
| Nilgun Cinar | cinarnilgun@gmail.com | Maltepe University Faculty of Medicine, Department of Neurology | 34844 | Istanbul | Türkiye |
| Miruna Florentina Ates | miruna.ates@gmail.com | Maltepe University Faculty of Medicine, Department of Neurology | 34844 | Istanbul | Türkiye |
| Sude Kendirli Aslan | drsudekendirli@gmail.com | Maltepe University Faculty of Medicine, Department of Neurology | 34844 | Istanbul | Türkiye |
| Berin Gulatar Turkoglu | berin_351@hotmail.com | Marmara University Pendik Training and Research Hospital, Neurology Clinic | 34899 | Istanbul | Türkiye |
| Ozum Yolcu | ozumtas@gmail.com | Mersin University Faculty of Medicine, Department of Neurology | 33200 | Mersin | Türkiye |
| Foksouna Sakadi | fokasaka@gmail.com | National Reference Teaching Hospital of N’Djamena | 190 | N’Djamena | Chad |
| Ilaha Azizova | dr.ilahaazizova@gmail.com | Neurological Clinic “New Technologies LTD” | AZ1009 | Baku | Azerbaijan |
| Esra Demir UNAL | md.esrademir@gmail.com | Nevsehir State Hospital Neurology Clinic | 50100 | Nevsehir | Türkiye |
| Marwa Yassien Badr | drmoroneuro@yahoo.com | Tanta University Hospital, Center of psychiatry, Neurology and Neurosurgery | 31527 | Tanta | Egypt |
| Elham Jafari | jafari4671@yahoo.com | Tehran University of Medical Sciences, Department of Neurology | 1417613151 | Tehran | Iran |
| Somayeh Nasergivehchi | snasergivehchi@gmail.com | Tehran University of Medical Sciences, Department of Neurology | 1417613151 | Tehran | Iran |
| Gizem Gursoy | drgizemgursoy@gmail.com | Umraniye Training and Research Hospital, Neurology Clinic | 34764 | Istanbul | Türkiye |
| Zeynep Selcan Sanli | zeynepsanlimd@gmail.com | University of Health Sciences, Adana City Training and Research Hospital, Neurology Clinic | 01230 | Adana | Türkiye |
| Fusun Mayda Domac | fusundomac@yahoo.com.tr | University of Health Sciences, Erenkoy Mental and Nervous Diseases Training and Research Hospital, Neurology Clinic | 34736 | Istanbul | Türkiye |
| Ayla Culha Oktar | aylaculha@yahoo.com | University of Health Sciences, Haseki Training and Research Hospital, Neurology Clinic | 34265 | Istanbul | Türkiye |
| Ahmet Kucuk | ahmetkucuk426120@gmail.com | University of Health Sciences, Konya Beyhekim Training and Research Hospital, Neurology Clinic | 42060 | Konya | Türkiye |
| Tuba Cerrahoglu Sirin | tubacerrahoglu@hotmail.com | University of Health Sciences, Sisli Hamidiye Etfal Training and Research Hospital, Neurology Clinic | 34396 | Istanbul | Türkiye |
| Muhammet Okay Orun | dr_m_okay@hotmail.com | University of Health Sciences, Van Training and Research Hospital, Neurology Clinic | 65300 | Van | Türkiye |
| Sibel Ozkan | ozkansybel@gmail.com | University of Health Sciences, Van Training and Research Hospital, Neurology Clinic | 65300 | Van | Türkiye |
| Mustafa Kiraz | musti_kiraz@hotmail.com | University of Health Sciences, Van Training and Research Hospital, Neurology Clinic | 65300 | Van | Türkiye |
| Gokhan Gorken | doktorazad@gmail.com | University of Health Sciences, Van Training and Research Hospital, Neurology Clinic | 65300 | Van | Türkiye |
| Pinar Gelener | drpinargelener@gmail.com | University of Kyrenia, Faculty of Medicine, Department of Neurology | 99300 | Kyrenia | Northern Cyprus |
